# One-Stage Hepatectomy for Bilateral Liver Metastasis After Portal Embolization: Tumor Behavior of the Non-embolized Contralateral Lobe

**DOI:** 10.7759/cureus.98786

**Published:** 2025-12-09

**Authors:** Sergio Riveros, Maria Irarrazaval, Antonio Peñailillo, Joaquin Hevia, Pablo Achurra, Nicolás Jarufe, Jorge Martínez, Luis Meneses, Martín Dib

**Affiliations:** 1 Department of Digestive Surgery, Pontificia Universidad Católica de Chile, Santiago, CHL; 2 Department of Interventional Radiology, Pontificia Universidad Católica de Chile, Santiago, CHL; 3 Department of Hepatobiliary Surgery, Hospital Clínico Universidad Católica, Santiago, CHL; 4 Department of Hepatobiliary and Pancreatic Surgery, Pontificia Universidad Católica de Chile, Santiago, CHL; 5 Division of Transplantation, Department of Surgery, Beth Israel Deaconess Medical Center, Harvard Medical School, Boston, USA

**Keywords:** bilobar metastasis, contralateral tumor, kgr, liver hypertrophy, portal vein embolization, pve, remnant tumor behavior

## Abstract

Background

Two-stage hepatectomy is a well-known strategy for bilobar liver metastases, “cleaning” the future liver remnant (FLR) in the first stage, using portal vein embolization (PVE), and performing a major hepatectomy in the second stage. We used an alternative approach, performing PVE, to continue chemotherapy and perform liver resection. We describe our experience emphasizing liver hypertrophy and the tumor behavior of the contralateral liver metastasis.

Methods

Non-concurrent cohort study. Patients who underwent PVE before liver resection for bilobar metastases were included. Pre-PVE variables, post-PVE volumetry, variation of metastasis diameter in the FLR, perioperative variables, and overall survival were evaluated.

Results

Fifteen patients were included. Neoadjuvant chemotherapy was given in 14 patients (93.3%). Median FLR pre- and post-PVE were 20.4% and 31.5%. The median degree of hypertrophy was 46.2%. Median kinetic growth rate was 1.9% per week. The metastasis diameter decreased or was maintained in 11 patients (73.3%) after PVE. Twelve (80%) underwent R0 resection. Major postoperative morbidity occurred in two patients (13.3%); no early mortality was reported.

Conclusions

One-stage hepatectomy after PVE effectively achieves an adequate FLR, with contralateral tumor growth absent in most cases. This allows the continuation of chemotherapy during the hypertrophy period and achieves R0 resection of bilobar metastasis.

## Introduction

The gold standard treatment for resectable colorectal liver metastases (CRLM) is surgical resection, as recommended in the latest National Comprehensive Cancer Network (NCCN) Guidelines (2024) and other guidelines [1]. Nevertheless, only 15%-20% of the patients with CRLM are candidates for surgical resection due to an insufficient future liver remnant (FLR). This situation increases the risk of postoperative hepatic dysfunction, overall morbidity, and mortality [2].

Portal vein embolization (PVE) is an alternative for stimulating hypertrophy of the FLR before major hepatic resections [1]. This procedure was first described in 1982 by Makuuchi et al. [3] and has been refined over time. PVE is frequently performed in patients with primary liver/biliary cancer and liver metastases, mainly from colorectal neoplasms [1,4]. It induces hypertrophy on one side of the parenchyma before a planned hepatic resection of the other side of the liver. It simultaneously becomes atrophic, allowing a safe surgical resection with negative margins and reduced perioperative morbidity and mortality in patients who achieve an adequate FLR.

Multiple factors are considered when deciding which patients would benefit from PVE, including baseline liver function, liver volumetry, and surgery complexity [4]. The NCCN 2024 treatment guidelines established a minimum FLR of 20% for patients without underlying disease to safely undergo hepatectomy. PVE must be considered in patients below this threshold [1]. Patients who underwent PVE before surgery to increase the FLR from less than 20% to greater than 20% had equivalent rates of liver failure to patients who did not require PVE [5-6].

However, several experimental and clinical studies reported tumor progression after PVE on FLR metastasis, which critically influences the subsequent management of these patients [2]. The underlying pathophysiological mechanism of tumor progression post-PVE has not been fully understood. In this context, the two-stage hepatectomy strategy was developed for bilobar liver metastases, doing PV embolization/ligation and “cleaning” the contralateral FLR as a first stage, and doing an ipsilateral major hepatectomy in a second stage.

This study aims to describe our institutional experience with PVE in patients with bilobar liver metastases who did not undergo contralateral lobe clearance prior to the procedure, followed by a one-stage hepatectomy. The primary objective is to evaluate the safety and oncologic outcomes of this strategy, with particular emphasis on the progression of tumors in the contralateral liver lobe during the interval between PVE and surgery.

## Materials and methods

Study design and population

**Figure 1:**
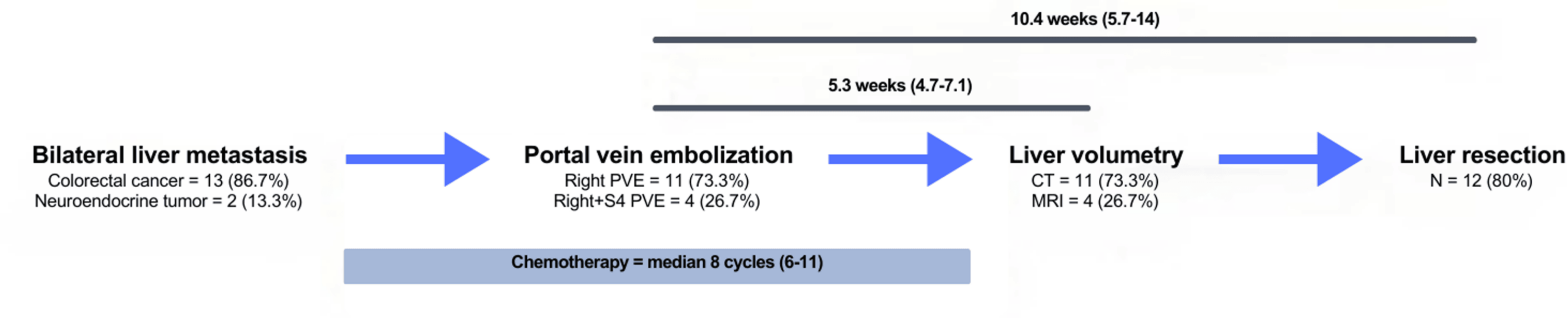
Scheme of the therapeutic approach Variables are in percentage or median (IQR) as appropriate. CT, computerized tomography; MRI, magnetic resonance imaging; PVE, portal vein embolization

This non-concurrent cohort study was conducted at Hospital Clínico Universidad Católica and approved by the Institutional Ethics Committee of Pontificia Universidad Católica de Chile, which granted a waiver of informed consent. Inclusion criteria comprised all patients with bilobar liver metastases, defined as having at least one lesion in the left lobe and one in the right lobe, who underwent PVE prior to liver resection between 2016 and 2020. The following data were recorded from the electronic medical record: gender, age, BMI, diagnosis, steatosis, neoadjuvant chemotherapy cycles and regimens, PVE-related variables (segment embolized, morbidity at 30 days), perioperative variables (operative time, transfusions, surgical procedure), postoperative variables (length of postoperative stay (LOPS), early complications, mortality). Liver steatosis was diagnosed on non-enhanced CT evaluation when the absolute liver Hounsfield units (HU) were less than 40 or the liver-minus-spleen difference was less than -10 HU [7]. For patients assessed with MRI, steatosis was determined using the proton density fat fraction (PDFF) method, with a PDFF value of 5% or greater indicating hepatic steatosis. A scheme of the therapeutic approach is shown in Figure 1.

Portal vein embolization

A multidisciplinary committee decided to perform PVE. Patients with an estimated FLR of less than 30% were candidates for PVE before hepatectomy. Embolization was performed under CT-guided vision. A trans-hepatic puncture was performed on the portal vein, and a 6 Fr venous access was installed; following venography, which segments to occlude was decided. PVE with segment IV branches was used when clinically feasible and indicated in patients anticipating segment IV resection. By selective catheterization, the corresponding segment was embolized using polyvinyl alcohol (PVA) 100-500-700 um, coils, and Onyx as occlusive materials. The transhepatic tract was closed with metal Nester coils. Complications were considered within 30 days of the procedure.

Liver volumetric analysis

All patients underwent formal liver volumetry based on CT or MRI imaging immediately before embolization and again three to six weeks after PVE, prior to hepatic resection. Volumetric analysis was performed using a semimanual segmentation technique with the Liver Analysis application included in IntelliSpace Portal version 9 (Philips Healthcare, Cleveland, OH). The percentage of FLR and maximum metastasis diameter in the FLR were calculated before and after PVE. The delta of metastasis diameter in the FLR was calculated using the difference in diameter before PVE and before surgery. The degree of hypertrophy (DH) was defined as the percentage point difference between the FLR volume before PVE and the FLR volume at final volume measurement, excluding non-functional structures. Kinetic growth rate (KGR) was calculated using the following formula: DH (%) / time elapsed from PVE to final volume assessment (weeks). Tumor progression was determined by an experienced radiologist through imaging analysis, evaluating changes in the longest diameter of the lesions on follow-up scans.

Outcomes and follow-up

Postoperative complications were considered within 30 days of surgery or during index hospitalization. Morbidity was reviewed based on the Clavien-Dindo Classification, and complications IIIa or more were categorized as major morbidity. Bile leak and posthepatectomy liver failure were defined using the International Study Group of Liver Surgery (ISGLS) classification [8,9]. Overall and disease-free survival (DFS) rates were evaluated in patients who underwent liver resection after PVE.

Statistical analysis

Statistical analysis was performed using SPSS software, version 25.0 (IBM SPSS Statistics for Windows, IBM Corp., Armonk, NY). Data are presented as median with interquartile range or as frequency and percentage, as appropriate. Overall survival (OS) and DFS rates were estimated using Kaplan-Meier survival curves.

## Results

A total of 15 patients were included. The median age was 67.6 years (IQR: 63.5-80.7), and 10 (67%) were female. The median BMI was 24.7 kg/m^2^ (IQR: 21.7-26.1) (Table 1).

**Table 1:** Demographic and preoperative variables CRC, colorectal cancer; IQR, interquartile range; NET, neuroendocrine tumor; PVE, portal vein embolization

	N (%)
Age; years (median, IQR)	67.6 (63.5-80.7)
Sex; female	10 (67%)
BMI; kg/m^2^	24.7 (21.7-26.1)
Diagnosis	
CRC metastases	13 (86.7%)
NET	2 (13.3%)
Liver steatosis	2 (13.3%)
Neoadjuvant chemotherapy	14 (93.3%)
Segment embolized	
Right PVE	11 (73.3%)
Right + segment IV	4 (26.7%)
Previous radiofrequency ablation	3 (20%)

The diagnosis was CRLM in 13 patients (86.7%) and neuroendocrine tumor in two patients (13.3%). Two patients (13.3%) had liver steatosis based on the imagological evaluation. Neoadjuvant chemotherapy was given in 14 patients (93.3%), with a median of eight cycles (range 6-11), and most of them (85.7%) received a regimen including oxaliplatin/5-fluorouracile (Table 1).

Eleven patients (73.3%) had right PVE alone, and four (26.7%) had right plus segment IV (S4) embolization. Three patients (20%) underwent radiofrequency ablation of metastases in the left lobe before PVE. One patient (6.7%) had a complication after PVE: metastasis bleeding on the embolized side, needing ICU medical support without the need for invasive treatment.

Pre- and post-PVE volumetry were done by computed tomography in 11 patients (73.3%) and magnetic resonance in four patients (26.7%). The median time between PVE and post-PVE volumetry was 5.3 weeks (IQR: 4.7-7.1).

Median FLR pre- and post-PVE were 20.4% (IQR: 17.4-28.8) and 31.5% (IQR: 25-40.5), respectively. The median DH was 46.2% (IQR: 35.6-72.4), and the median KGR was 1.9% per week (IQR: 1.1-2.6).

Metastases in the liver remnant were localized in segments II-III in 10 patients (66.7%) and segment IV in five patients (33.3%). Median metastasis diameter in the FLR pre- and post-PVE was 18 mm (IQR: 11-22) and 12 mm (IQR: 8-14), respectively. This diameter was maintained or decreased in 11 patients (73.3%), with a median delta of 5 mm (IQR: 2-10).

In four patients, the diameter of contralateral metastases increased after PVE (26.7%). All four patients had neoadjuvant chemotherapy between PVE and post-PVE imaging. Three were considered resectable after PVE (diameter increased by 13, 15, and 15 mm, respectively). Two of them were resected, and one declined liver resection during the early COVID-19 pandemic. One patient was deemed non-resectable due to tumor growth in the FLR (the patient had four tumors in FLR; the diameter increased only by 6 and 14 mm on the largest ones after PVE, but he also had insufficient liver growth with FLR < 30%). 

In total, only three patients could not undergo liver resection (n = 3, 20%), one of them due to evidence of extrahepatic pulmonary metastases before surgery, one of them declined, and one of them (1/15; 6.7%) secondary to tumor growth in FLR and insufficient FLR growth (described above). All three patients had neoadjuvant chemotherapy.

Twelve patients (80%) underwent liver resection. The time between PVE and hepatectomy was 10.4 weeks (IQR: 5.7-14). In one patient, a rescue associating liver partition and portal vein ligation surgery (ALPPS) was decided at the time of surgery, doing parenchymal transection and performing the second stage hepatectomy seven days later. Right trisectionectomy was performed in 50% (n = 6), right trisectionectomy plus caudate in 8.3% (n = 1), right extended hepatectomy (partial S4 resection) in two (16.7%), right hepatectomy in three (25%), and resection of the non-embolized remnant tumors in nine patients (60%). The median operative time was 330 minutes (IQR: 240-420). Blood transfusions were required in six (40%) patients, all less than three units (Table 2).

**Table 2:** Surgical and postoperative variables IQR, interquartile range; PVE, portal vein embolization ^a^Clavien-Dindo classification

	N (%)
Liver resection	
Yes	12 (80%)
No	3 (20%)
Time between PVE and hepatectomy; weeks (median, IQR)	10.4 (5.7-14)
Surgery	
Right trisectionectomy	6 (50%)
Right trisectionectomy + caudate	1 (8.3%)
Right extended hepatectomy	2 (16.7%)
Right hepatectomy	3 (25%)
Operative time; minutes (median; IQR)	330 (240-420)
Requirements of blood transfusions	6 (40%)
Early postoperative complications^a^	4 (26.7%)
I-II	2 (16.7%)
III or more	2 (16.7%)
Length of stay; days (median, IQR)	6 (5-8)

Early postoperative major morbidity occurred in two patients (13.3%). One patient required an unplanned surgery due to evisceration, one patient (6.7%) had post-hepatectomy liver failure, no patients had biliary leaks, and no 90-day mortality was reported. The median length of postoperative stay was six days (IQR: 5-8) (Table 2).

The median follow-up was 25.6 months (IQR: 5.8-51.3). The OS rate at 24, 36, and 48 months from PVE was 85.7%, 72%, and 58%, respectively. The DFS rate at 12, 22, and 48 months from PVE was 66.7%, 58%, and 50%, respectively.

## Discussion

Liver resection is the treatment of choice for primary and metastatic liver tumors, and R0 resection is a major prognostic factor [1]. In our series, the diameter of the non-embolized liver metastasis decreased in 73.3% of the patients after PVE, and 12 patients (80%) underwent R0 liver resection with adequate FLR hypertrophy (median 46.2%). 

In the past, more than half of CRLM and other metastases were deemed unresectable due to the inadequate FLR [2]. Major liver resection in patients with small FLR can increase morbidity and mortality, mainly due to postoperative liver failure [1]. PVE procedure allows safe surgical resection with negative surgical margins and low perioperative mortality in patients with inadequate FLR. Our group considers the use of PVE for FLR <30% by volumetric estimation of patients who underwent chemotherapy, which is similar to that considered by other groups [2,4], according to guidelines that considered PVE in anticipated FLR ≤20% in normal livers, ≤30% in chemotherapy livers and ≤40% in cirrhotic livers [4,10]. It has been described that 4.3% of patients will present insufficient FLR post-PVE [11]. In our study, five patients still had FLR<30% after PVE; nonetheless, they were all resected by waiting a more extended time (four patients), and one patient in whom it was decided to perform a rescue ALPPS procedure with a favorable postoperative outcome.

Radiofrequency in the non-embolized segment, part of the FLR, was used in 20% of this series to control the disease in the FLR and prevent local tumor progression during neoadjuvant therapy and liver hypertrophy post-PVE. We also consider a single-stage surgery.

According to the available evidence, different techniques are used for PVE without a single standardized approach. There are various methods, materials used, and rates of extension to segment IV, without apparent differences in the DH [10]. We use PVA coils and onyx as embolization material for the ipsilateral approach to avoid vascular damage to FLR and facilitate access to segment IV [1,4]. Portal pressure should be measured during the procedure, especially in patients with cirrhosis. Severe portal hypertension with a portosystemic pressure gradient greater than 12 mmHg is a relative contraindication for major liver resection, and the indication for PVE needs to be reconsidered. PVE has been associated with major bleeding complications, including hemoperitoneum, subcapsular hematoma, hepatic artery injury or pseudoaneurysm, and transient haemobilia. The most typical morbidity described secondary to PVE is portal thrombosis and intestinal ileus, and in PVL, hepatic abscesses, with no difference in specific liver morbidity [11]. In this study, we report only one post-PVE complication in a patient with metastatic hemorrhage needing transfusion and medical treatment without the need for invasive or surgical procedures. 

The DH is variable in those with liver disease; in this series, we describe only two patients with moderate liver steatosis and none with liver cirrhosis. Our results are similar to those reported by other groups, with an increase in FLR of around 10% and a DH between 40 and 45%, including other malignant etiologies [11-13]. The use of concurrent chemotherapy is an important issue due to the hepatotoxic effects of many of these drugs. Reported studies consider the use of chemotherapy around PVE treatment in a high percentage of included patients [12,14], like our series, where 93% (14/15) used chemotherapy peri-PVE with a median length of eight cycles. A study comparing FLR hypertrophy four weeks after PVE in patients with CCR liver metastasis with and without chemotherapy (42 vs. 22, respectively), including patients with Bevacizumab, showed comparable results on hypertrophy rates [15].

Similarly, other groups demonstrated that chemotherapy does not influence liver hypertrophy, maintaining considerable efficacy in preventing disease progression [11,13,16]. Chemotherapy has shown a significantly lower rate of disease progression, with clear survival benefits in surgical and non-surgical patients [17,18]. Therefore, chemotherapy has not been shown to affect FLR hypertrophy, and maintaining systemic therapy during FLR growth allows the selection of those patients with more aggressive tumor biology, giving an advantage of PVE alone vs. a two-stage open approach in which chemotherapy may need interruption [12]. 

Several studies are comparing the outcomes of strategies to increase liver hypertrophy, including portal vein occlusion techniques such as PVE and portal vein ligation (PVL), two-stage hepatectomy (TSH), and the association of hepatic partition and PVL for staged hepatectomy (ALPPS). A recent meta-analysis showed no significant differences between PVE and PVL regarding increased FLR, morbidity, 30-day mortality, and disease progression [11]. The ALPPS procedure has shown a faster FLR growth rate than PVE but with the disadvantage of higher morbidity and mortality, which could even affect DFS as a long-term oncological outcome [12]. Due to the minimally invasive characteristics of the PVE technique, which are associated with similar results in FLR, it is recommended as a preferred strategy in patients with inadequate FLR before major hepatectomy.

One of the concerns that hepatobiliary surgeons may have about not “cleaning” the contralateral lobe after PVE is the potential accelerated growth of contralateral tumor lesions. Although PVE is a safe procedure, it has been associated with tumor progression in embolized and FLR lobes [14,19-21]. Current evidence suggests that portal flow obstruction stimulates positive regulation of cytokines and growth factors, which, added to the compensatory increase in hepatic arterial flow, could induce and stimulate tumor pathways for their expansion [1,2,12]. However, the evidence based on systematic reviews is still tiny and controversial. From our study, 80% of the patients underwent surgery with complete tumor resection, similar to 70% reported by other groups [2,4,12,13]. In a study of 450 patients undergoing PVE for CCR metastasis, 30% did not proceed to liver resection, mainly due to tumor progression (24.6%), two-thirds intrahepatic, and one-third with extrahepatic progression [12]. A study that evidenced the growth of post-PVE CCR metastases found no correlation between tumor volume growth and FLR increase [13]. For indications of PVE in primary liver tumors, the conclusions about subsequent tumor growth are even less clear [1,2,4,10-13]. Even two-stage hepatectomy in the series reported 20% of drop-outs due to tumor disease progression [22,23]. This disease progression may be secondary to pre-existing tumor growth, novel tumor formation, or the activation of dormant micro-metastases [12]. A smaller number of studies reported tumor behavior of the non-embolized remnant; one showed a 17% tumor volume growth compared to 4% in the non-embolized segment [24].

In our study, most patients (11/15; 73.3%) had decreased or stable size of the dominant lesion of the non-embolized liver lobe after PVE and were all resected. Four patients (26.7%) had contralateral tumor growth post-PVE, although three of them were still resectable. Only one (6.7%) patient was considered non-resectable due to tumor growth in the FLR, associated with poor tumor biology while on chemotherapy, as well as insufficient liver hypertrophy. One could argue that this patient could have undergone a two-stage hepatectomy, first cleaning the left lobe, but likely, his tumor biology would have precluded the second stage. Further prospective studies and evidence are needed to clarify the behavior of metastases in the non-embolized liver segments following PVE. 

The proposed strategy in this study allows the selection of those patients with worse tumor biology determined by disease progression after PVE while maintaining systemic treatment with chemotherapy, and it may avoid an unnecessary first-stage surgery with interruption of chemotherapy in patients who may progress and not be able to complete a second-stage. For patients with good tumor biology, this strategy decreases the time between PVE and hepatectomy [14,25-27], and it can be combined with other procedures such as trans-arterial chemoembolization (TACE) and radioembolization of the hepatic artery. Further studies are needed to demonstrate efficacy and safety, but it may be a promising strategy [2,26].

After hepatectomy, major morbidity is reported in 25% [13] and early mortality in 3.8% [11]; this last one is mainly determined by liver failure (32.8%). In our study, we describe two patients (13.3%) with major morbidity and no early mortality. Post-hepatectomy liver failure in patients who achieve adequate preoperative FLR is reported around 10% [12]. Our study has only one case (6.7%). The DFS rate at 22 months was 58%, and the OS rate at 48 months was 58%, respectively, which are considered favorable oncological outcomes considering their short expected survival without R0 resection. 

This study has several limitations that must be acknowledged. First, it is a retrospective analysis with a small cohort of 15 patients, which limits the statistical power and generalizability of the findings. Second, there is an inherent selection bias, as the study includes only patients who underwent PVE with the intention to proceed to hepatectomy, rather than patients selected from a broader diagnostic cohort. These limitations should be taken into account when interpreting the results and drawing clinical conclusions.

## Conclusions

One-stage hepatectomy after PVE alone is safe and effective in achieving an adequate FLR for patients with bilateral liver metastases. Despite being a retrospective study with a small sample size, this approach allows the continuation of chemotherapy during the hypertrophy period without favoring accelerated growth of tumor lesions in the non-embolized contralateral lobe in most cases, and achieves R0 resection of bilobar metastases with only one liver surgery in 12 (80%) of patients. A two-stage hepatectomy to “clean” the contralateral lobe first to avoid remnant tumor growth may not be justified in most patients. Prospective studies are necessary to understand the best approach when using PVE for bilobar disease.
